# Development of Down Syndrome Research Over the Last Decades–What Healthcare and Education Professionals Need to Know

**DOI:** 10.3389/fpsyt.2021.749046

**Published:** 2021-12-14

**Authors:** Karin Windsperger, Stefanie Hoehl

**Affiliations:** ^1^Division of Obstetrics and Feto-Maternal Medicine, Department of Obstetrics and Gynecology, Medical University of Vienna, Vienna, Austria; ^2^Research Unit Developmental Psychology, Department of Developmental and Educational Psychology, Faculty of Psychology, University of Vienna, Vienna, Austria

**Keywords:** Down syndrome, trisomy 21, developmental outcome, phenotypic heterogeneity, Alzheimer's disease, medical comorbidities, social environment, prenatal counseling

## Abstract

Down syndrome (DS) is the most prevalent neurodevelopmental disorder, with a known genetic cause. Besides facial dysmorphologies and congenital and/or acquired medical conditions, the syndrome is characterized by intellectual disability, accelerated aging, and an increased likelihood of an early onset Alzheimer's disease in adulthood. These common patterns of DS are derived from the long-held standard in the field of DS research, that describes individuals with DS as a homogeneous group and compares phenotypic outcomes with either neurotypical controls or other neurodevelopmental disorders. This traditional view has changed, as modern research pinpoints a broad variability in both the occurrence and severity of symptoms across DS, arguing for DS heterogeneity and against a single “DS profile.” Nevertheless, prenatal counseling does not often prioritize the awareness of potential within-group variations of DS, portraying only a vague picture of the developmental outcomes of children with DS to expectant parents. This mini-review provides a concise update on existent information about the heterogeneity of DS from a full-spectrum developmental perspective, within an interdisciplinary context. Knowledge on DS heterogeneity will not only enable professionals to enhance the quality of prenatal counseling, but also help parents to set targeted early interventions, to further optimize daily functions and the quality of life of their children.

## Introduction

Down syndrome (DS) is the most common neurodevelopmental disorder with known genetic causes, and an incidence of 1 in 691 live births (1). This suggests that ~417,000 people with DS live in Europe (2). Currently, an expansive menu of prenatal diagnostic methods for DS is spreading worldwide, advancing the diagnosis of DS from postnatal to prenatal (3). Giving an expectant parent a fetal diagnosis of DS provides them with 2 options: keeping or terminating their pregnancy, following the lack of a cure (4).

Prenatal counseling is crucial for providing parents with an accurate picture of DS so that informed decisions can be made in the context of their own beliefs and values (3). Although studies are still examining the nature of DS, portraying the expected neurodevelopmental outcomes of affected children remains challenging. Indeed, retrospective studies indicate that parents felt that the information received during prenatal counseling was inaccurate, outdated, and unbalanced, and either too negative or too optimistic (5–7). Without appropriate professional training or updated professional development regarding the individual variability in outcomes associated with DS, prenatal counselors might present expectant parents with inaccurate information or impressions. Therefore, expectant parents may not receive the level of information needed. Accordingly, all professionals working with families affected by DS must be aware of the most current scientific research regarding the heterogeneity of phenotypic outcomes (8).

This mini-review closes an existent literature gap by providing a concise update on the available information on within-group variations in the DS phenotype of infants, children, and adolescents for professionals. First, a gross outline of DS research is given, focusing on the significant paradigm shift from a group- to an individual-level approach. Second, the current knowledge on significant within-group variations of DS in cognitive, behavioral, emotional, and olfactory functioning is summarized. Finally, the review concludes by arguing that only an interdisciplinary approach allows for the description of realistic individual DS profiles. The scope of this review is to further increase the awareness on DS heterogeneity concerning developmental outcomes.

## A Paradigm Shift in DS Research: From a Group- to Individual-Level Approach

DS research dates back to 1866, when the English physician John Langdon Down systematically described the syndrome for the first time (9, 10). In addition to intellectual disability (ID), he chronicled a distinct physical phenotype of individuals with DS, conjecturing that they were “born to the same family” (page 9) (10, 11). The century following his pioneering work was filled with publications of diverse medical case studies documenting a range of physical traits and medical comorbidities, leading to various etiologies (10, 11).

Almost 100 years later, the French pediatrician and cytogeneticist, Jérôme Lejeune, identified the genetic basis of DS in 1959 as an extra copy of all or part of chromosome 21 (10, 12). The discovery of “trisomy 21” paved the way for further research, to elucidate genotype-phenotype-relationships (13, 14). Since its original description, classical DS research has analyzed the syndrome's phenotypes relative to neurotypicals and/or other neurodevelopmental disorders, hence providing group-level data that have advanced our basic knowledge of DS (8). It is characterized by both typical physical features that make the syndrome “instantly recognizable” (page 8) and ID (11). Common appearance includes craniofacial dysmorphologies, short stature, low muscle tone, and a proportionally large tongue. Additionally, medical comorbidities, such as sleep apnea, visual and/or hearing problems, congenital heart defects, and altered behavioral, hematopoietic, endocrine, gastrointestinal, neurological, and musculoskeletal conditions, are linked to DS (10).

Most of these medical problems are treatable with pharmacotherapy and/or surgical interventions. Therefore, among the key focuses in recent DS research is the widespread field of neurocognition, associating DS with weaknesses in motor ability, auditory processing, verbal short-term memory, and expressive language. However, relative strengths in visuospatial processing, receptive language, and some aspects of social functioning have been reported (15–18). Further, DS is associated with accelerated aging and an increased likelihood of the early onset of Alzheimer's disease (AD) (18).

Although the generalizability of the characteristics of DS has been questioned repeatedly in the history of DS research, the group-level approach is a long-held standard (19, 20). However, this traditional view has changed, following a growing number of studies, which pinpoint significant within-group variations across individuals with DS at many levels of description. Pioneer studies have launched this paradigm shift, from a group to an individual-level approach, by highlighting significant individual differences in genetics, cell biology, brain research, and subsequently, parts of cognitive research on DS [see (8)]. These studies suggest that this heterogeneity may be continued in DS phenotypes (8). The following review aims to supplement the prevailing knowledge about the variability of the developmental outcomes of DS by addressing this issue from an interdisciplinary and applied science perspective, as this practical information may be the most useful for professionals to pass to expectant parents.

## Infants, Children, and Adolescents With DS: Variability in Developmental Outcomes

### Acquisition of Developmental Milestones

Generally, it was assumed that infants and children with DS reached developmental milestones in the same linear fashion as their non-DS peers, but at later chronological ages. This view is too simplistic, as the age of acquiring milestones among infants and children with DS is reported to vary significantly (21, 22). For example, the mean age at the onset of babbling is ~15 months, with an interindividual variability of 10 months. Similarly, sphincter control is acquired by DS children at an approximate age of 44 months, with 22 months of interindividual variability (22). Notably, Locatelli et al. suggested that the age at which developmental milestones are reached influences the subsequent development of diverse cognitive domains significantly (21, 22).

### Intellectual Disability (ID)

ID, defined by an intelligence quotient (IQ) score of <70, is reported to be universal in the DS population. However, this construct presents in DS with large interindividual variability (23). The majority of individuals with DS fall within the severe (IQ 20–35) to mild (IQ 50–69) range of ID. However, some cases reach IQ scores equivalent to children without ID (14, 24). Research on the developmental trajectories of cognitive function in neurotypicals shows that IQ is a construct that remains relatively stable and consistent across ages. A slight decline was observed only in older adults (14). Conversely, DS research has identified a linear decline in IQ scores as development progresses, starting in the first year of life (i.e., cognitive gains do not keep pace with chronological age). Notably, single IQ levels and the degree of cognitive decline vary across the DS group (14).

### Language

Language is another cognitive domain that generates significant differences among individuals with DS. DS is associated with weaknesses in expressive language and a relative strength in the receptive language (18). The available literature reports developmental delays in both language domains, becoming apparent no later than age five, yet with wide individual differences (25, 26). Regarding vocabulary acquisition and growth, longitudinal studies reported an existing continuum, ranging from non-verbal children to those with a vocabulary close to the normal range (27, 28). Children with DS use gestures as a means of communication, which has been positively associated with the development of spoken vocabulary (29). Nevertheless, significant individual variability in the extent to which this “gestural advantage” is used has been demonstrated by empirical data (30). All within-group differences in language development persist into adulthood (26).

### Memory

Memory and learning deficits are universal characteristics of DS and are known to become more pronounced as development progresses (14). In classical DS research, the findings of affected memory domains are mixed, suggesting underlying variability (18). Indeed, scientific data demonstrate that there are individual differences in both implicit and explicit memory (8, 31). Regarding the latter, significant within-group variations are described for short-term verbal and long-term visual memory (8). Individuals with DS often show deficits in processing local detail. Therefore, classical DS literature claims that individuals with DS were “global processors.” However, this preference for global over local processing does not always occur in the DS population. Therefore, individuals with DS cannot be simply categorized into one of these processing styles (32).

### Executive Function (EF)

EF encompasses a range of cognitive processes involved in goal-oriented behavior, and is a domain in which individuals with DS are shown to have pronounced difficulties (33). The areas of working memory, attention, planning, and inhibition are considered particularly challenging for individuals with DS; emotional control is considered a relative strength (34, 35). However, significant individual differences in EF across the DS group have become evident (33, 36). Within-group variations in auditory attention have been identified *via* electrophysiological measurement among toddlers with DS, data that also predict differences in language abilities as development progresses (37). Patterns of executive dysfunction appear to be relatively consistent across development until adulthood (23, 34).

### Adaptive Behavior (AB)

Children and adolescents with DS are known to be severely impaired in AB, which subsumes behavioral skills that enable them to function independently in their everyday life (23, 38). Generally, AB encompasses 4 domains: socialization, communication, daily living, and motor skills (23). Significant within-group variations were apparent for all the 4 domains. For example, DS has been associated with sociability, friendliness, affection, empathy, good competence in forming relationships, and high tendency to smile (39). Yet, children and adolescents with DS are also considered stubborn, to show little accommodation to social partners, and approach strangers inappropriately (40). Some individuals with DS have even deficits in socialization to the extent of a comorbid diagnosis of autism (41).

### Maladaptive Behavior (MB) and Psychiatric Comorbidities

MB encompasses a range of behaviors that impede an individual's activities of daily living or the ability to adjust to and participate in particular settings (23). Approximately 1/4 to 1/3 of individuals with DS exhibit clinically significant levels of maladaptive behavioral concerns (42–44). This behavioral construct is another domain that yields significant within-group differences (21, 23, 45). More difficulties with “anxious-depressed” symptoms are observed among adolescents than younger children with DS (23). Children with DS often exhibit externalizing behavior (46). The manifestation of MB is significantly higher when neurobehavioral disorders are concomitant (47–49). According to the available literature, the manifestation of psychiatric features, including autism, depression, and the attention-deficit/hyperactivity disorder, vary significantly, between 6 and >50% (42, 44, 50, 51). Channell et al. underscored within-group differences in the behavioral domain by subtyping a >300-person DS group, hence identifying a separate “behavioral” class as described in Table 1 (23).

**Table 1:** Characterization of the 3-class model of individuals with DS (*N* = 314; 6–25 years) based on the variability observed in cognitive and behavioral measures, identified by Channell et al. (23) using a latent profile analysis.

	**3-class model proposed by Channell et al**. **(****23****)**		
	**Normative class**	**Cognitive class**	**Behavioral class**
Number of participants	*N* = 153 (48%)	*N* = 109 (35%)	*N* = 52 (17%)
Strengthens (relative to sample average)	Cognitive skills (IQ, visuospatial abilities), adaptive behavior, executive function	–	Cognitive skills (IQ, visuospatial abilities), adaptive behavior
Weaknesses (relative to sample average)	–	Cognitive skills (IQ, visuospatial abilities), executive function, adaptive behavior, maladaptive behavior (ASD, hyperactivity)	Maladaptive behavior (ASD), executive function

*ASD, autism spectrum disorder; IQ, intelligence quotient*.

### Emotional Functioning

The emotional profiles of individuals with DS have remained underexplored, which could be attributed to the assumed stereotype of high sociability in this population (52, 53). Available literature provides variable data about whether children and adolescents have difficulties in emotional functioning (52). Whereas, some studies negate differences in identifying basic emotion in faces between DS and non-DS groups, other scientific reports indicate that children and adolescents with DS have impairments in this emotional skill [see Roch et al. (52)] (54–57). Deficits in recognizing facial expressions were not generalized to all emotions, but mostly to fear (52, 58). Other studies report impairments in determining feelings, including surprise, anger, and neutral expression (40, 58–61). Some studies pinpoint problems in ascertaining negative emotions (40). Moreover, an inability to distinguish between fear and sadness is another atypical pattern that has been reported among some individuals (58). Most of these deficits are identified during infancy and childhood. Therefore, a negative impact on the subsequent development of interpersonal relationships is discussed (52). As previously mentioned, studies have exclusively gathered data at the group level. Moreover, further research should examine whether inconsistencies in findings across studies can be attributed to underlying within-group variations.

### Olfactory Functioning

The number of studies on olfactory function among patients with DS is limited and relatively out of date (62–69). Historical studies have described olfactory deficits in the DS population for many years (62, 63, 65, 70). Because rhinologic pathologies have been ruled out by studies showing nasal function in DS as comparable to controls, central-nervous causes are suggested (64). More recently, Cecchini et al. described olfactory function as severely impaired among adults with DS (71). They found a positive correlation between odor identification and cognition (71). To date, the largest study, which included people with DS and under 18 years, described a minimal impairment of olfactory functioning among children and adolescents (9–17 years), which became pronounced in young adulthood (18–29 years) and was the lowest in adulthood (30–50 years) (72). Of the three groups, DS, IQ, and age-matched controls, significant within-group differences were evident only in the DS group (72). However, large and detailed analyses of olfactory function in light of within-group variations among children and adolescents with DS are still lacking. Odor identification deficits are considered a valid non-invasive early marker of AD. Therefore, future research on whether olfactory dysfunction can help to ascertain the subset of children and adolescents with DS that will later develop AD is warranted.

### Alzheimer's Disease (AD)

Although the issue of AD appears outside the scope of this review, the following considerations must be made when the heterogeneity of DS is discussed with expectant parents from a full-spectrum developmental perspective. Owing to a shared genetic predisposition, individuals with DS have an increased likelihood of developing early onset AD in adulthood (18). Prevalence rates of dementia among the DS population vary significantly in the literature, from 8 to 100% (18, 73). Recent brain research has identified Alzheimer's plaques among some children with DS, that is, as early as 8 years of age, whereas some DS brains show no plaques until early adulthood (14, 26). Although AD neuropathology occurs in virtually all individuals with DS over the age of 30, only a subset of people develop clinical symptoms of dementia (26, 74, 75). Hence, it is apparent that the widespread interindividual variability, typical for DS, is a pivotal feature not only during development, but also during aging (26). Aging is part of the continuous lifespan development. Accordingly, some authors argue that AD should be considered a disease that occurs during development, rather than aging (76).

## Extrinsic Influencing Factors of Developmental Outcomes of Infants, Children, and Adolescents With DS

### Medical Comorbidities

In addition to cognitive limitations, parents must be informed that there is a list of medical comorbidities associated with DS. Some of them, including congenital heart defects (CHD), seizures, visual and/or hearing impairments, autism, and sleep disruptions, are known to moderate cognitive functioning (18). Analogous to neurodevelopmental outcomes, both the occurrence and expression of congenital and/or acquired medical complications are variable (18). For example, 41–56% of infants with DS are born with a CHD, with an atrioventricular septal defect that occurs between 31 and 61% being the most common form (77, 78). Cognition, gross motor skills, and language are significantly worse among infants with DS and CHD, relative to peers without CHD, in some, but not in all related studies (79–81). For example, Alsaied et al. showed that children with DS and CHD, who undergo cardiac surgery during their first year, have no significant differences in neurodevelopmental outcomes at preschool and school age. However, as infants and toddlers, they were prone to poorer outcomes in receptive, expressive, and composite language compared to children with DS without CHD, suggesting that deleterious effects may be dependent on clinical management (82).

### Home Environment

Another variable that affects the observed variability of DS phenotypes, which is influenced by the expectant parents, is the home environment. According to Karmiloff-Smith et al., the genetic syndrome changes the family context in terms of parent-child-interactions (8). D'Souza et al. demonstrated that parental depression, a disease linked to difficulties in responding to the child in a sensitive and consistent manner, explained deficits in expressive language development among children between 8 and 48 months of age with DS (83). Similarly, there is evidence that vocabulary development among children with DS is influenced by how parents respond to their children's communication. Deckers et al. argued that mothers with a higher level of education had a better ability to fine-tune their communication with their children with DS (28). Further demographic factors, including socioeconomic status, neighborhood demographics, and the availability of therapeutic resources, modulate the developmental outcomes of DS effectively (84, 85). These data demonstrate that only an interdisciplinary approach that considers psychological, physical, and social parameters will enable professionals to accurately inform expectant parents on how the DS phenotype will be expressed in each individual.

## Discussion

Although DS has been examined for a long time, that is 155 years, it is still one of the least understood genetic ID syndromes. The most significant reason for this is the high degree of phenotypic variability observed in the DS population, an issue that professionals are often unaware of when discussing the diagnosis with expectant parents. However, DS research has advanced from a group to an individual-level approach, attempting to acknowledge within-group differences at many levels of basic science (8). To expand on this wealth of data, this mini-review has shed light on the available information on individual variability in the developmental outcomes of infants, children, and adolescents with DS from an applied science perspective, which will enhance the quality of prenatal counseling. Diverse developmental domains, including cognition, behavior, and emotional and olfactory functioning, have been discussed.

The evaluation of developmental outcomes from a full-spectrum perspective, however, must not only address different developmental domains, but also the change of phenotypes over time (86). Outcome variables are not completely intact or impaired uniformly throughout development, but manifest as variations at an early state, that may be magnified with age, ending up as either a strength or a weakness. Therefore, parents should be made aware that early development can be considered a critical window of opportunity to set adequate phenotype-specific interventions before deficits become severely pronounced (87). Thus, the maximization of individual potential is possible. In addition to psychological factors, other influencing variables must be considered by parents when the variability of DS phenotypes is discussed. According to Karmiloff-Smith who states that having a neurodevelopmental disorder changes both the social environment and physical status, only an interdisciplinary research approach can successfully describe valid profiles of individuals with DS (8).

The most convincing argument for emphasizing individual variability among DS groups and discussing them with expectant parents are both an average life expectancy of 60 years combined with an early onset of Alzheimer's disease in the DS population (18). Focusing on individual differences in the development of DS may be the best approach for exploring the risk and protective factors of AD (88, 89).

Modern DS research shows that developmental heterogeneity has become increasingly validated (23). Moving forward, these up-to-date data must be disseminated under the supervision of professionals so that prenatal counseling can be optimized in quality, hence allowing parents to gain realistic expectations about the future of their children. Thus, more targeted treatments and interventions can be set to improve the daily function and quality of life.

## Author Contributions

KW and SH designed the paper. KW did the literature research and wrote the manuscript. SH provided intellectual input and critically revised the manuscript. Both authors contributed to the article and approved the submitted version.

## Conflict of Interest

The authors declare that the research was conducted in the absence of any commercial or financial relationships that could be construed as a potential conflict of interest.

## Publisher's Note

All claims expressed in this article are solely those of the authors and do not necessarily represent those of their affiliated organizations, or those of the publisher, the editors and the reviewers. Any product that may be evaluated in this article, or claim that may be made by its manufacturer, is not guaranteed or endorsed by the publisher.
